# Diagnostic Role of Prostate-Specific Membrane Antigen in Adrenocortical Carcinoma

**DOI:** 10.3389/fendo.2019.00226

**Published:** 2019-04-16

**Authors:** Yulin Gu, Weijun Gu, Jingtao Dou, Zhaohui Lu, Jianming Ba, Jie Li, Xiaocong Wang, Hongyan Liu, Guoqing Yang, Qinghua Guo, Li Zang, Kang Chen, Jin Du, Yu Pei, Yiming Mu

**Affiliations:** ^1^Department of Endocrinology, Chinese People's Liberation Army General Hospital, Beijing, China; ^2^Department of Pathology, Chinese People's Liberation Army General Hospital, Beijing, China

**Keywords:** adrenal tumor, adrenocortical carcinoma, immunohistochemical staining, prostate-specific membrane antigen, diagnostic tool

## Abstract

**Objective:** To investigate the role of PSMA in the differential diagnosis of adrenocortical carcinoma samples (ACCs) and adrenocortical adenoma samples (ACAs), to validate the prognostic role of PSMA in patients with ACCs, and to explore the possibility that this marker can differentiate localized ACCs from adrenal metastases from other sites.

**Methods:** PSMA protein expression in tissue samples from 50 ACCs, 90 ACAs (including 20 from patients who presented with Cushing's syndrome, 20 aldosterone-producing adenomas and 50 non-functional tumors) and 10 tissues that were metastases from other primary sites was assessed by immunohistochemistry. The clinical and pathological characteristics were compared, the intensity and density were analyzed, and the prognostic role was evaluated.

**Results:** The analysis of clinical and pathological features revealed that the size of ACCs was greater than that of benign tissues and the ACC patients were older than the ACA patients (*p* < 0.01). The percentage of PSMA-positive vessels, the mean intensity and the degree of staining density were found to be significantly lower in ACAs than in ACCs (*p* < 0.01). In these 140 samples, 60% of the ACCs were grouped in the positive category. The samples were negative for metastases that were from other primary sites. The ENSAT stage and Ki-67 were correlated with PSMA expression. The survival distribution revealed that high PSMA expression did not show any prognostic relevance in the current ACCs series. Those samples with a score of > 3.5 were 75 times more likely to be malignant (OR = 75). We established a cut-off score of 3.5 (*p* < 0.05), which had 46% sensitivity and 99% specificity. Paralleling PSMA and Ki-67 maximized the area under the curve, with 72% sensitivity and 100% specificity.

**Conclusions:** Our results strongly confirm that PSMA is helpful for distinguishing benign from malignant tumors and that its high expression levels correlate with a high ENSAT stage and high proliferation. The combination of PSMA and Ki-67 can be particularly useful. Furthermore, PSMA might be a useful tool for the identification of localized adrenal carcinoma and metastatic carcinoma.

## Introduction

Adrenocortical tumors are frequent (2–3% of the general population according to autopsies), and most are found incidentally. ACC is an uncommon and aggressive cancer arising in the cortex of the adrenal gland. The incidence of ACC has been estimated at 0.7–2 cases per 1 million persons, accounting for 0.02% of all cases of carcinoma per year. Furthermore, even with surgery, 75–85% of patients will experience a relapse (1–3), and the prognosis is poor. The main clinical challenge is to properly identify ACCs among the majority of adrenocortical adenomas (4–6). The Weiss (7) criteria are used to diagnose ACC in adults; however, in some cases, the diagnosis of malignancy is not straightforward. Moreover, even when malignancy is easily assessable, differential diagnosis of ACC originating from primary adrenal or extra-adrenal neoplasms is difficult. Adrenal metastases from other sites are relatively infrequent but can be a source of diagnostic error, particularly with small biopsy samples. As a consequence, many diagnostic tools have been proposed for diagnosing ACC, several of which are based on specific marker immunodetection and gene, micro-RNA, or gene methylation profiling. Among the most promising novel tools, PSMA has been recently proposed as a sensitive marker.

PSMA (also known as folate hydrolase 1, glutamate carboxypeptidase II, and N-acetyl-L-aspartyl-L-glutamate peptidase I) is a 100-kDa type II transmembrane glycoprotein receptor heterogeneously expressed at low levels in the normal prostate secretory epithelium but is expressed homogenously at considerably higher levels in prostate carcinoma (8, 9) and in the endothelium of most solid tumor neovasculature (8–11). Because of its unique expression pattern, which is limited to tumor-associated endothelial cells, PSMA may also be an interesting molecule for vascular targeting. Recently, PSMA has been identified as a potential diagnostic and therapeutic target in ACCs because it is highly expressed in carcinomas (12, 13), and Zr89-J591 WB-PET-CT was reported to be useful for detecting metastases. Furthermore, PSMA expression increases directly with increases in the tumor grade, stage, and adverse clinical outcome (14–16).

To date, PSMA expression in solid tumor neovasculature has been reported for only a limited number of tumor types and in a limited number of cases. The aim of the present study, therefore, was to investigate whether PSMA can be used to classify ACCs and ACAs. The study also aimed to compare the clinical and pathological features in different expression of PSMA and to validate its prognostic role in patients with ACC. Finally, this study explored the possible use of this marker to differentiate between localized ACCs and metastases from other sites.

## Materials and Methods

### Tissue Collection

A retrospective review of a prospectively maintained tissue bank was performed to identify patients who had undergone surgery for adrenocortical tumors at the Chinese People's Liberation Army (PLA) General Hospital between June 2005 and June 2016. All procedures were in accordance with the Helsinki Declaration and approved by the medical ethics committee of the Chinese PLA General Hospital. Only tumors arising from the cortex of the adrenal gland and from patients older than 18 years of age were included in this study. Ten tissues that were metastases from other primary sites were also included. Adrenal medullary tumors were excluded. The pathological diagnosis of ACC was confirmed using Weiss criteria and biological characteristics, and scores of 3 or higher were considered to indicate malignancy. Demographic, clinical and pathological data were collected for all the patients from electronic medical records. A total of 150 cases were included in this study: 50 ACCs, 90 ACAs (including 20 from patients who presented with Cushing's syndrome, 20 aldosterone-producing adenomas and 50 non-functional tumors) and 10 tissues that were metastases from other primary sites. For all cases, clinical, pathological, and follow-up data were collected. All studies were performed with approval from the Chinese Public Liberation Army General Hospital.

### Immunohistochemical Staining for CD34 and PSMA

To assess the expression of PSMA within the vascular endothelium, we also stained for an endothelial cell marker, CD34, to identify the vasculature. Haematoxylin and eosin-stained slides of the 150 tissue samples were reviewed. The tissue sections (4-μm-thick) were mounted on glass slides, deparaffinized and rehydrated. The slides were stained for CD34 (1:200; ZM-0046; Origene) and PSMA (diluted 1:100; ab19071; Abcam). The proliferative index was evaluated in parallel with Ki-67 (1:200; ZM-0166; Origene).

For antigen retrieval, the samples underwent treatment in a pressure cooker for 1 min at 125°C in EDTA buffer (pH 8.0) and were then cooked for another 2 min and 30 s at 100°C. The samples were allowed to cool to room temperature before incubation with 3% hydrogen peroxide for 15 min. Subsequently, the sections were incubated overnight at 4°C with primary antibody and then incubated sequentially with secondary antibody (Zhong Shan Jin Qiao) and diaminobenzidine for 20, 20, and 10 min, respectively. Finally, the slides were counterstained with haematoxylin (Abcam), dehydrated and mounted. The appropriate positive controls were prepared.

### Staining Interpretation and Staining Intensity

All immunohistochemical analyses were performed under a multihead microscope by 2 endocrine pathologists. We calculated the percentage of PSMA compared to CD34. We chose 2 areas within each slide possessing the highest concentration of microvessels stained with haematoxylin and eosin and used CD34 to count the microvessels in each of these hotspots. The mean count of these areas for each stain was recorded. The expression of PSMA in tumor vessels was evaluated by identifying the same hotspots used for the analysis of CD34, which was expressed in 100% of the microvascular tissue on each slide. The staining intensity was graded on a 0-3 scale as follows: 0, no staining; 1, weak; 2, moderate; and 3, strong. These values were then placed into quartiles (0%, 0; 2–25%, 1; 26–50%, 2; 51–75%, 3; and 76–100%, 4) (Supplementary Figures 1, 2) and added to the PSMA intensity to obtain a composite PSMA staining score. A composite score of 0, 1–3, or >3 was considered to represent no staining, weak staining or strong staining, respectively. Prostate tissue was used as a positive control.

All cases were categorized as “negative/low” or “high” expression according to the mean score value of the overall population.

### Statistical Analysis

The clinical and pathological characteristics were compared with the staining patterns of the antibodies using Fisher's exact or χ2 and Student's *t*-tests for categorical and continuous variables, respectively. To analyse the prognostic impact of all clinical and pathological variables considered, a univariate overall survival analysis was performed based on the Kaplan-Meier product limit estimate of survival distribution. Unadjusted differences between survival curves were tested using the log-rank test. Statistical significance was set to *p* < 0.05. All tests were performed using GraphPad Prism version 7.0 for Mac and SPSS version 21 (Microsoft).

## Results

### Patient Demographics

A total of 150 samples, including 20 CPAs, 20 APAs, 50 NFTs, 50 ACCs and 10 tissues that were metastases from other primary sites, were analyzed in this study. The tumor size and age were found to differ significantly between ACCs and benign tissues (*p* < 0.01) (Table 1), whereas gender and location were not found to be different.

**Table 1:** Clinical and pathologic features of ACA and ACC cases.

**Parameter**	**ACC**	**ACA**			***p***
		**CPA**	**APA**	**NFT**	
M/F ration	28/22	4/16	13/7	25/25	0.3
Age, mean ± SD (y)	46 ± 13 (19–72)	48 ± 13 (23–69)	48 ± 6 (35–57)	52 ± 9 (28–72)	<0.05
Size, mean ± SD (cm)	11 ± 4.8 (1.5–22)	3.7 ± 2 (2–9)	1.8 ± 1 (1–5.5)	2.9 ± 0.8 (1.5–5.5)	<0.01
L/R ration	23/27	10/10	12/8	25/25	0.5
ENSAT stage	1–2: 16	-	-	-	-
	3–4: 34	-	-	-	-
FU	AWD: 5	-	-	-	-
	DOD: 26	-	-	-	-
	NED: 7	-	-	-	-
	lost to FU: 12	-	-	-	-
Median overall survival (mo)	53 ± 39	-	-	-	-

*ACC, adrenocortical carcinoma; ACA, adrenocortical adenoma; CPA, cortisol-producing adenoma; APA, aldosterone-producing adenoma; NFT, non-functional tumor; M, male; F, female; L, left; R, right; FU, follow-up; AWD, alive with disease; DOD, died of disease; NED, no evidence of disease (Student t-test)*.

### PSMA Protein Expressions

**Figure 1:**
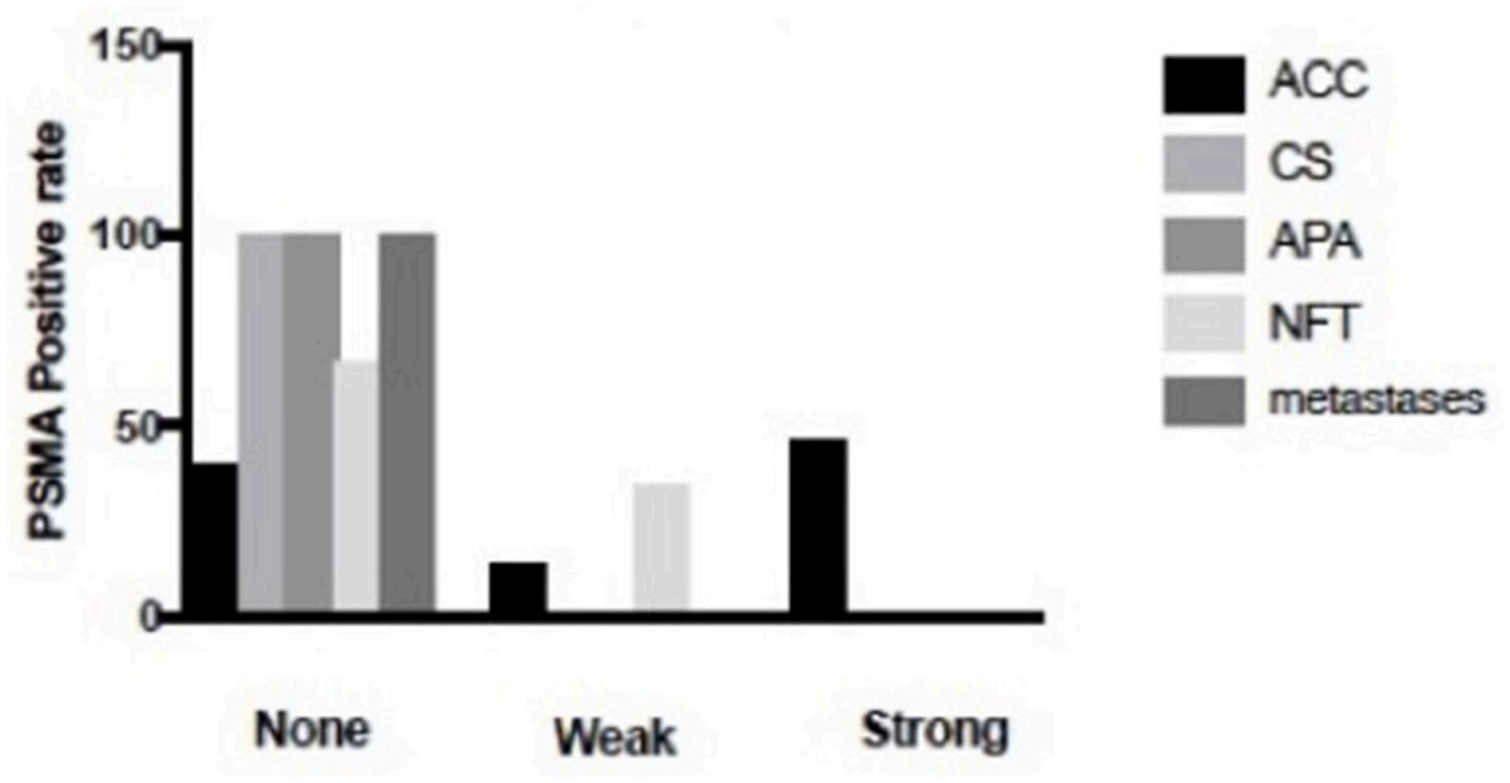
Percentage of composite PSMA scoring intensity of 150 cases. Of the 50 ACC cases, 40% of them were negative for staining, 14% of them were weakly stained, 46% of them were strongly stained. In contrast, 34% of the NFT was weakly stained, 66% of NFT was stained negative. What is more, 100% of CS, 100% of APA and 100% of metastases were negative for staining.

**Figure 2:**
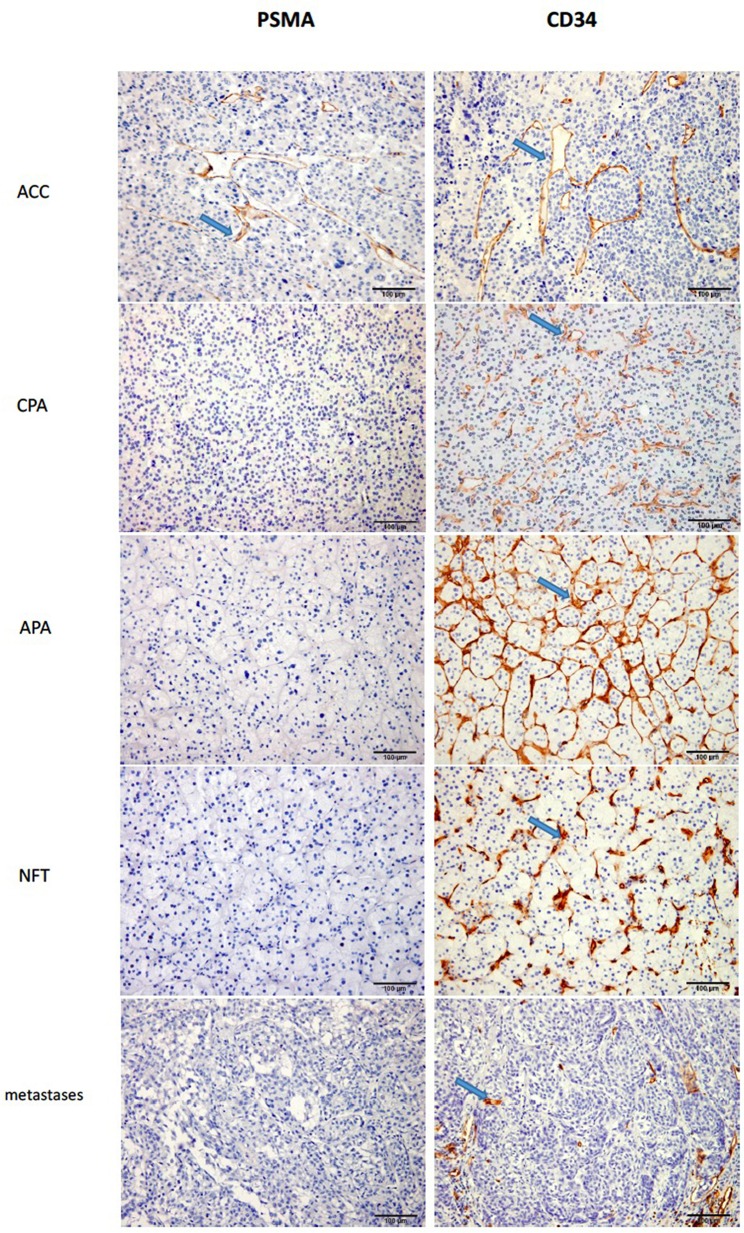
The staining of PSMA and CD34 in ACA and ACC (X200). As we can see from the figure, the staining of PSMA in CPA, APA, NFT and metastases were negative, while in ACC it was positive with high percentage.

The percentage of PSMA-positive vessels was significantly lower in ACAs (19%, 1.0 ± 1, *p* < 0.01) than in ACCs (60%, 0.3 ± 0.7) (Figures 1, 2). Positive PSMA expression was observed on the neovascular endothelial cells of tumors. The mean intensity of PSMA among ACC (1.5, 0–2) cases was notably higher than that among ACA (0. 0–0) cases, and the difference was statistically significant (*p* < 0.01). When considering the degree of staining density, ACC (1.5, 0–3) cases demonstrated significantly higher scores than ACA (0, 0–0) cases (*p* < 0.01). In these 140 samples, 40% of the ACCs did not stain, 14% stained weakly and 46% stained strongly. When parallel Ki-67 index and PSMA staining, the positive rate increased to 76%. In contrast, 81% of ACAs showed no staining, and 19% presented weak staining. No difference in the PSMA staining intensity was found between different types of benign tumors.

The results showed that no staining with the antibody was observed in tumors that were metastases from other primary sites (Table 2).

**Table 2:** Relationship between different tumor types.

**Parameter**	**PSMA**	
	**Median (Quartile)^a^**	***p***
ACC vs. ACA	3(0–5) vs. 0(0–0)	<0.01
ACC vs. CPA	3(0–5) vs. 0(0–0)	<0.01
ACC vs. APA	3(0–5) vs. 0(0–0)	<0.01
ACC vs. NFT	3(0–5) vs. 0(0–2)	<0.01
ACC vs. metastases	3(0–5) vs. 0(0–0)	<0.01
CPA vs. APA	–	–
CPA vs. NFT	0(0–0) vs. 0(0–2)	<0.01
CPA vs. metastases	–	–
APA vs. NFT	0(0–0) vs. 0(0–2)	<0.01
APA vs. metastases	–	–
NFT vs. metastases	0(0–2) vs. 0(0–0)	<0.01

a *Total score. The rank sum test was used*.

### Correlation of PSMA With Clinical and Pathologic Variables

We compared the gender, age, size, location, ENSAT stage, disease status and Ki-67 index values between the negative/low-staining and high-staining samples (Table 3). The results showed that the ENSAT stage and Ki-67 value were correlated with PSMA expression. Consequently, we can infer that PSMA is a prognostic indicator. However, the survival distribution showed no association between high PSMA expression and overall survival in the current ACC series, but there exists a decreasing tendency of lifetime among high PSMA expression. Those samples with scores >3.5 were 75 times more likely to be malignant than benign (OR = 75). Therefore, we established a cut-off score of 3.5 (*p* < 0.05), which had 46% sensitivity and 99% specificity. Paralleling PSMA and Ki-67 maximized the area under the curve, with 72% sensitivity and 100% specificity.

**Table 3:** PSMA immunohistochemistry: clinical and pathologic correlates in ACC.

**Parameter**		**PSMA**		
		**Negative/low**	**High**	***p***
Sex	M	15	13	1.0
	F	12	10	
Age^a^	≤46 y	13	10	0.7
	>46 y	14	13	
Size (cm)^a^	≤11	16	11	0.5
	>11	11	12	
Side	L	13	13	0.5
	R	14	10	
ENSAT stage	1–2	14	2	<0.01
	3–4	13	21	
Disease status	AWD: 5	2	3	-
	DOD: 26	14	12	
	NED: 7	5	2	
Ki-67 index	<20	13	3	<0.01
	≥20	14	20	

a *Median value*.

*Fisher's exact or χ2 test was used*.

## Discussion

ACC is a rare neoplasm with poor prognosis, and the main prognostic factor is the tumor stage. The differential diagnosis between ACCs and ACAs is crucial and relies on the Weiss score, which has some drawbacks. Weiss's study also analyzed the expression of several cell cycle proteins, including Ki-67, bcl-2, p53, mdm-s, p21 and p27, but a significant predictive value for metastatic disease could not be identified. To overcome the limitations of the Weiss score, many researchers have recently searched for immunohistochemical markers of malignancy, prognostic factors and therapeutic targets (5, 17–19). In the present study, we investigated the diagnostic role of PSMA in a large series of ACCs.

The clinical and pathological correlates between ACC and ACA patients showed significant differences in size and patient age. The size of ACCs (11 ± 4.8) was clearly greater than that of ACAs, and patients with metastases were older than those with other tumors. The explanation for these differences in tumor size and patient age is that most of the patients with ACCs were asymptomatic but had adrenal incidentaloma that led to the discovery of the ACCs during treatment. Additionally, the biological characteristics of carcinoma may be responsible for the difference. The median overall survival time was 53 ± 39 months, which is the same as that reported in other studies.

Recently, Conway RE et al reported that LQ, the dipeptide product of PSMA cleavage of LQE (laminin peptides), efficiently activates endothelial cells *in vitro* and enhances angiogenesis *in vivo*. These results characterize a novel PSMA substrate, provide a functional rationale for the upregulation of PSMA in cancer cells and he tumor vasculature and suggest that the inhibition of PSMA could lead to the development of new angiogenic therapies (20). In our study, 40% of the neovasculature of ACCs were grouped in the negative category, and 60% stained positive, and no difference was found between ACAs. Our results from a large number of cases confirmed that PSMA was more highly expressed at the protein level in carcinoma than in neoplasm. Furthermore, compared with the negative cases, the masses with a composite score >3.5 were 75 times more likely to be malignant than benign, and a cut-off score of 3.5 exhibited 46% sensitivity and 99% specificity. Paralleling PSMA and Ki-67 maximized the area under the curve, with 72% sensitivity and 100% specificity. Crowley (12) also studied PSMA in 16 normal adrenals (NML), 16 ACAs and 15 ACCs and revealed that 83% of ACCs showed moderate to high staining, and a cut-off score of 4 exhibited high specificity and sensitivity. Our positive rate is lower than that found by Crowley, and the reasons for this difference may include (a) the difference in the antibody used; (b) the different lab conditions; (c) the small number of ACCs (only 15 were analyzed in the previous study, which may inevitably cause bias, whereas we included a large number of ACCs in our study); and d) the difference in the ethnicity of the study subjects. Most of the previous studies were conducted among Americans or in populations from Western countries. Our study is the first to have been conducted in China.

Because PSMA has been reported to be present in the endothelium of tumor vessels, we used CD34 for comparison and found a significant decrease in the vascular density in ACCs compared with that of ACAs. Bernini et al, Diaz Cano et al, and Michael et al. drew the same conclusion (19, 21, 22), whereas Xu et al. reported an increase in the microvascular density of ACCs relative to that of benign tumors (23).

We found that the metastases could not be stained. Most of the previous studies investigated metastases from the primary cancer to the lymph nodes or to other organs, but few of them studied adrenal metastases from other points. Our results revealed that PSMA might be a useful marker for distinguishing metastases and primary adrenal cancer from metastases in other organs, but further study is required, and the mechanism requires additional research.

Finally, our results showed no association between PSMA expression and overall survival, but there exists a decreasing tendency of lifetime among high PSMA expression. Methodological differences (scoring system and cut-off value selection, size of the cases, patient characteristics) are possibly responsible for the differences between our results and those reported by Michael J. P. Crowley (12). In addition, the differences between the results obtained in these two studies are due to the differences in the treatments after surgery. In primary gastric adenocarcinoma and primary colorectal adenocarcinoma, no association has been found between PSMA expression and overall survival (8). Because the ENSAT stage and Ki-67 score were correlated with PSMA expression, we can speculate that a high level of PSMA might predict high tumor stage and a poor prognosis.

In our study, we analyzed a large number of cases that revealed the diagnostic and therapeutic role of PSMA. However, the numbers of patients in the follow-up of our study was not sufficient. In total, 38 patients were followed up, corresponding to a rate of 76%, which might indicate an impact on the association between PSMA expression and overall survival.

Taken together, our findings showed that PSMA is expressed in the neovasculature of adenocarcinoma. Furthermore, our results strongly confirm that PSMA is helpful for distinguishing a benign from a malignant tumor. Paralleling PSMA and Ki-67 can be particularly useful. High expression levels of PSMA correlate with a high ENSAT stage and high proliferation. Finally, PSMA is not expressed in metastatic carcinoma in the adrenal gland; thus, PSMA might be a useful tool for identifying localized adrenal carcinoma or metastatic carcinoma.

## Ethics Statement

All procedures performed in studies involving human participants were approved by the medical ethics committee of the Chinese PLA General Hospital and in accordance with the 1964 Helsinki Declaration and its later amendments or comparable ethical standards. This article does not describe any studies with animals performed by any of the authors. Informed consent was obtained from all individual participants included in the study.

## Author Contributions

YG did the experiment, analyzed the data. She was a major contributor in writing the manuscript. JL, XW, and HL supervised histological examination. JingD, ZL, JB, GY, QG, LZ, KC, JinD, and YP made substantial contributions to conception and design, or acquisition of data, or analysis and interpretation of data, and have been involved in revising it critically for important intellectual content. WG and YM were the superior advisors. They given final approval of the version to be published, and agreed to be accountable for all aspects of the work in ensuring that questions related to the accuracy or integrity of any part of the work are appropriately investigated and resolved. All authors read and approved the final manuscript.

### Conflict of Interest Statement

The authors declare that the research was conducted in the absence of any commercial or financial relationships that could be construed as a potential conflict of interest.
