# Supplementary material for: Diagnostic Role of Prostate-Specific Membrane Antigen in Adrenocortical Carcinoma
Source: Front Endocrinol (Lausanne). 2019 Apr 16;10:226. doi: 10.3389/fendo.2019.00226 (PMC6476981; doi:10.3389/fendo.2019.00226)
Supplement: Supplementary file 1 [file Data_Sheet_1.docx]

**PSMA CD34**


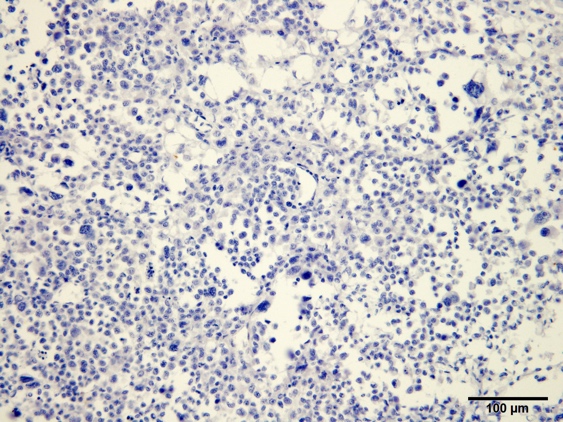

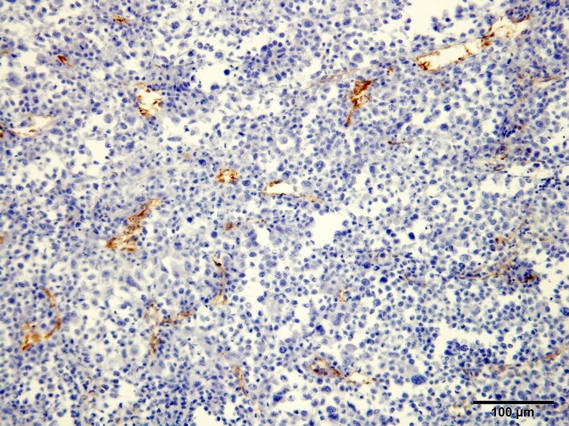


Negative


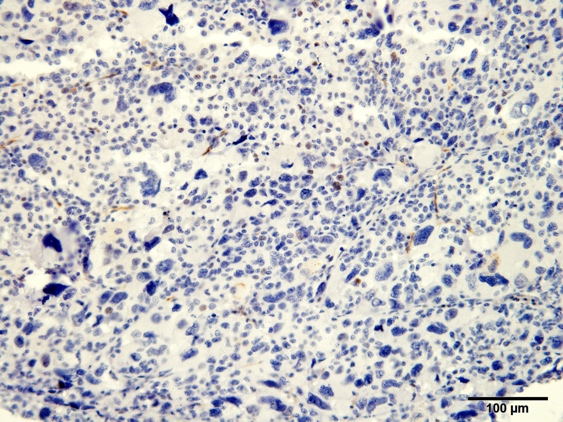

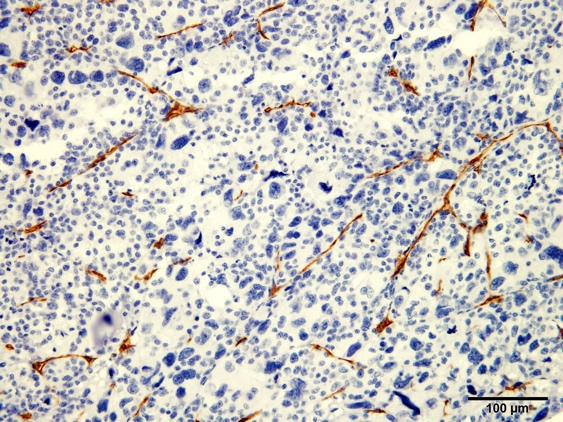


Score 1

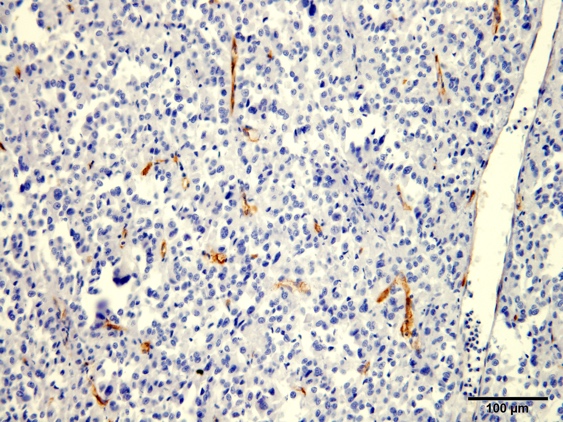

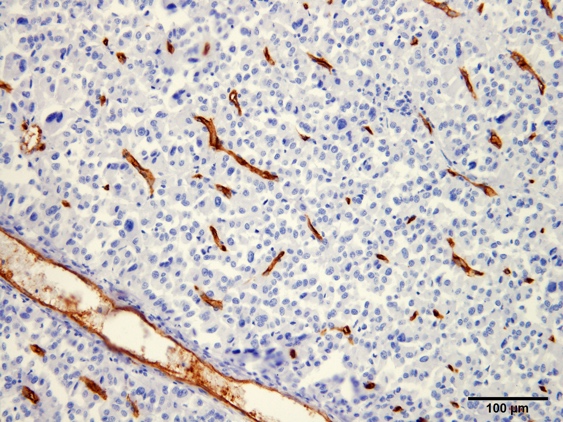


Score 2


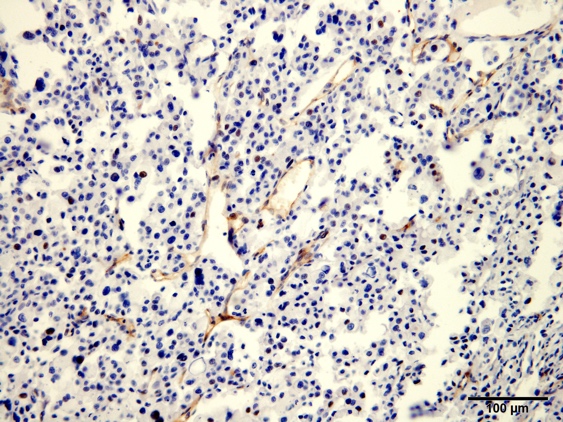

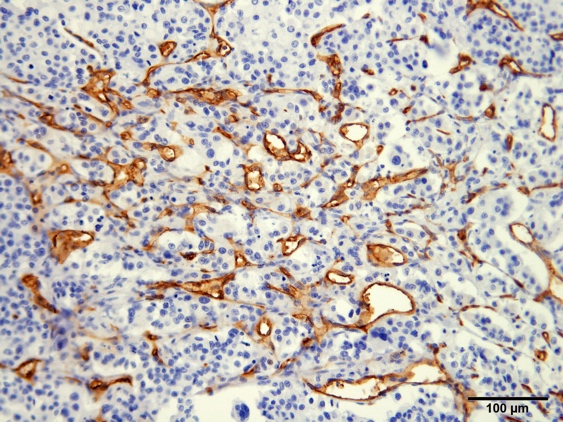


Score 3


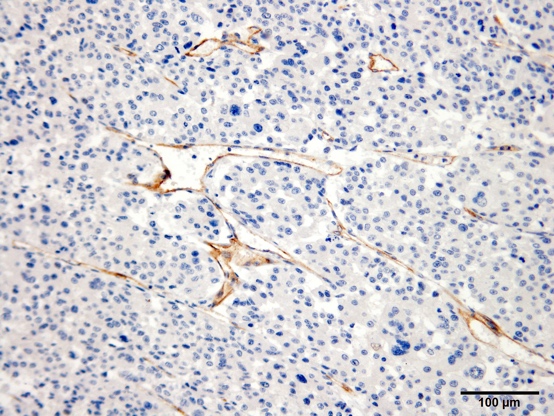

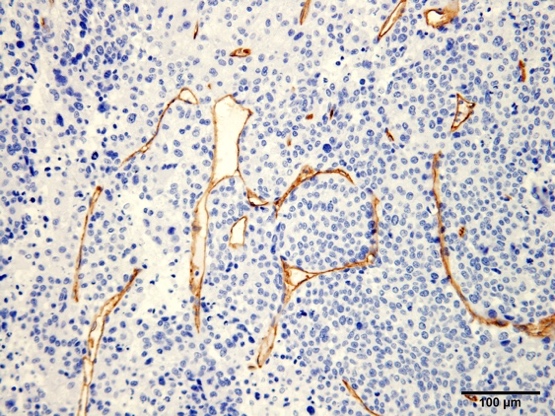


Score 4

**Supplementary Figure 1. The staining grade of PSMA and CD34 in ACC (X200).** Score 0: 0%; score 1: 26%-50%; score 2: 26%-50%; score 3: 51%-75%; score 4: 76%-100%.
