# Supplementary figures and images for: Diagnostic Role of Prostate-Specific Membrane Antigen in Adrenocortical Carcinoma
Source: Front Endocrinol (Lausanne). 2019 Apr 16;10:226. doi: 10.3389/fendo.2019.00226 (PMC6476981; doi:10.3389/fendo.2019.00226)

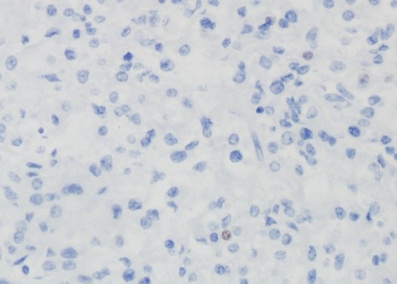

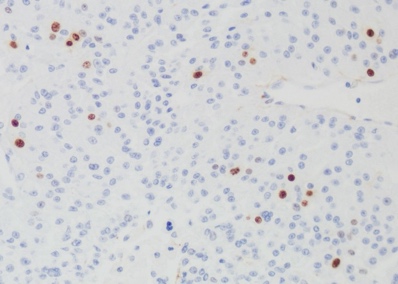

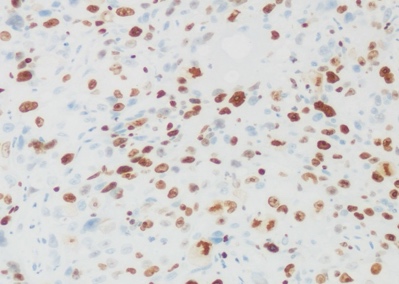


**C**

**B**

**A**

**Supplementary Figure 2. The staining grade of Ki-67 (X200).** A: Score 0; B: Score <20%; C: Score ≥20%

Supplement: Supplementary file 2 [file Data_Sheet_2.docx]
